# Outcomes of a model integrating tuberculosis testing into COVID-19 services in South Africa

**DOI:** 10.4102/phcfm.v14i1.3709

**Published:** 2022-12-13

**Authors:** Chipo Mutyambizi, Lynne Wilkinson, Kate Rees, Shabir Moosa, Tom Boyles

**Affiliations:** 1 Anova Health Institute, Johannesburg, South Africa; 2 International AIDS Society, Johannesburg, South Africa; 3 Centre for Infectious Epidemiology and Research, Faculty of Health Sciences, University of Cape Town, Cape Town, South Africa; 4 Department of Community Health, School of Public Health, University of the Witwatersrand, Johannesburg, South Africa; 5 Department of Family Medicine, Faculty of Health Sciences, University of the Witwatersrand, Johannesburg, South Africa; 6 Right to Care NPC, Johannesburg, South Africa; 7 London School of Hygiene and Tropical Medicine, London, United Kingdom

**Keywords:** COVID-19, tuberculosis, South Africa, clinical algorithm, primary health care

## Abstract

The coronavirus disease 2019 (COVID-19) pandemic led to a reordering of healthcare priorities. Health resources were turned to the screening and diagnosis of COVID-19, leading to a reduction in tuberculosis (TB) testing and treatment initiation. An innovative model that integrated TB and COVID-19 services was adopted at primary care facilities in Johannesburg Health District, Gauteng. This short report illustrates results from this model’s implementation in two facilities. Patients were screened for COVID-19 at a single point of entry and separated according to screening result. Self-reported human immunodeficiency virus (HIV) status, symptom, and symptom duration were then used to determine TB risk amongst those screening positive for COVID-19. Data from clinical records were extracted. Approximately 9% of patients with a positive symptom screen (*n* = 76) were sent for a TB test and 84% were sent for a COVID-19 test. Amongst those sent for a TB test, 8% (*n* = 6) had TB detected, and amongst those sent for a COVID-19 test, 18% (*n* = 128) were positive. Amongst those with COVID-19-related symptoms, 15% (*n* = 130) presented with a cough or fever and were known HIV positive and 121 (93%) of these were sent for a COVID-19 test and 31 (24%) were sent for a TB test. Given the HIV prevalence and symptoms in our study, our results show lower-than-expected TB tests conducted.

**Contribution:**

Our study documents the outcomes of an innovative way to combine operational workflows for TB and COVID-19. This provides a starting point for countries seeking to integrate TB and COVID-19 screening and testing.

## Introduction

Until the emergence of severe acute respiratory syndrome coronavirus 2 (SARS-CoV-2), the virus that causes coronavirus disease 2019 (COVID-19), in 2020, tuberculosis (TB) caused the highest number of infectious disease-related deaths globally^1,2,3^ with estimated 10 million cases in 2019. South Africa has a population of 59.3 million and has one of the highest TB incidences in the world with 852 cases per 100 000 population in 2018.^4^ The country also has the highest COVID-19 burden in Africa with 4 million tests confirmed and reported and over 102 000 deaths as at 28 August 2022.^5^ Since the emergence of SARS-CoV-2, there has been a reduction in TB testing and treatment initiation.^1^ The Stop TB partnership estimates that nine of the countries with the highest TB cases experienced an average of 23% decline in TB diagnosis and treatment initiation in 2020.^6^ The COVID-19 pandemic led to a reordering of healthcare priorities from both the demand and supply sides.^7^ Movement restrictions because of lockdowns and fear of contracting COVID-19 at health facilities impeded access to healthcare, including TB services. COVID-19 prioritisation at health facilities also resulted in limited services for other healthcare needs. It was, therefore, important to develop effective models of care that accounted for both diseases and mitigate the impact of COVID-19 on TB services. South Africa’s TB case finding is reliant on a TB screening approach in which patients are requested to self-report any traditional TB symptoms. To ensure continued TB screening after the emergence of COVID-19 and given the overlap in TB and COVID-19 symptoms, TB testing in the model described in this study was considered in people with a positive COVID-19 screen.

## Integrated tuberculosis/COVID-19-testing model

An integrated primary care facility approach to ensure appropriate testing for TB in patients’ screening positive for COVID-19 was developed^8^ and implemented in six pilot/model primary care facilities and expanded to more than 100 thereafter within Johannesburg Health District, Gauteng. For this study, two facilities were selected out of the six facilities in which the study was implemented. The COVID-19 and TB primary care facility screening assessments were developed in April 2020 by a team of public health, family medicine and infectious diseases specialists. A detailed description of the model is provided elsewhere^8^ along with online-training materials.^9^ Briefly, the model applied color-coding zones within clinics to inform patients and clinic staff of their entry or exit zone.^10^ Patients were rapidly screened for COVID-19 by lay workers at a single point of clinic entry using the following screening questions. In the past 14 days, have you developed any of the following: cough, fever, shortness of breath, sore throat, loss of sense of smell or taste or worsening of a chronic cough? (the yellow zone). Patients’ screening negative proceeded to normal clinic services with enhanced infection prevention and control (IPC) measures, including routine TB services for those reporting chronic symptoms (blue zone). Patients’ screening positive proceeded to a separate section of the facility (the orange zone), typically set up outside the clinic building using gazebos, and tents with full contact and respiratory IPC precautions.

Patients in the orange zone were assessed by a clinician, with severely ill patients being referred to a higher level of care. For all the other patients, the need for COVID-19 testing was then determined using updated person under investigation criteria. Oropharyngeal, nasopharyngeal or sputum sample was collected for diagnosing COVID-19.

**FIGURE 1:**
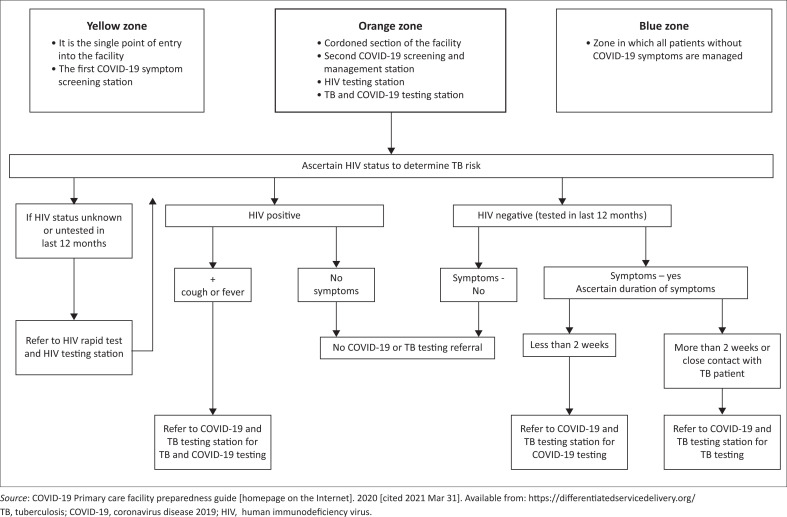
Algorithm for integration of COVID-19, HIV and tuberculosis testing amongst mild-to-moderately ill patients screening positive for COVID-19 in primary health care clinics.

The clinician then ascertained the HIV status of all patients in order to determine TB risk (see Figure 1^10^). Patients with an unknown human immunodeficiency virus (HIV) status (untested in the last 12 months) were offered HIV testing. Patients with a known self-reported HIV-positive status and with cough or fever were classified as at risk for TB and received sputum-based testing with Xpert^®^ MTB/RIF Ultra. Patients with a known HIV-negative test result in the last 12 months and who reported TB symptoms for more than two weeks or reported close contact with a TB patient were also classified as at risk for TB and received sputum-based testing. Details of patients who had a COVID-19 PCR test conducted or TB were recorded in a paper register.

## Outcomes of the model at two facilities in Johannesburg

This study was conducted at two purposively selected community healthcare centres Alexandra (Alexandra township) and Stretford (Orange Farm township) in Johannesburg Health District, Gauteng Province, South Africa.

Data were collected retrospectively from paper registers and the National Health Laboratory Service laboratory information system. A clinical note form and COVID register were developed for the purposes of rapid model implementation. The clinical note forms were completed for all patients entering the orange zone, and the COVID-19 register was completed for all patients who had a COVID-19 test. All available clinical stationaries for patients visiting the orange zone between May 2020 and July 2020 were captured. These months fall within a period in which the country experienced its first wave of COVID-19.

A total of 2217 records for patients who had a COVID-19 test and 846 clinical notes for patients who visited the orange zone (positive COVID-19 symptom screen) were captured into Research Electronic Data Capture (REDcap) database.^11^ Of the 846 notes, 473 were female patients (56%) and out of the nine symptoms checked for the most common symptom reported was a cough, which was reported by 608 patients (72%). A total of 113 patients (13%) did not report back any symptoms. Forty percent (*n* = 293) of those with a captured symptom had a recorded symptom duration; of these, 9% (*n* = 26) had a symptom duration of more than two weeks. Forty-five percent of patients were known to be HIV negative (*n* = 377), 20% were known HIV positive (*n* = 168), 35% reported an unknown HIV status on initial assessment (*n* = 297) and four patients had no data for HIV status captured. We cannot report HIV results for those with unknown status, as test results were not recorded on the same form. Amongst those with COVID-19-related symptoms, 15% (*n* = 130) presented with a cough or fever and were known HIV-positive and 121 (93%) of these were sent for a COVID-19 test and 31 (24%) were sent for a TB test. Data from the clinical forms show that approximately 76 (9%) patients with a positive COVID-19 symptom screen were sent for both a TB and COVID-19 test, 3 were sent for a TB test only and 635 (75%) were sent for a COVID-19 test only. Amongst those sent for a TB test, 8% (*n* = 6) had TB detected and all were bacteriologically confirmed. Amongst those sent for a COVID-19 polymerase chain reaction (PCR) test, 18% (*n* = 128) were positive. Amongst those sent for both a COVID-19 and TB test, none came back positive for both.

### Ethical considerations

Ethical approval was provided by the Human Sciences Research Council (HSRC) (No. REC 3/22/08/18), and permission was obtained from the Johannesburg District Research Committee.

## Discussion

Our study describes outcomes of a simple, innovative model that integrated TB and COVID-19 diagnosis at primary health care clinics in the early stages of the COVID-19 pandemic in South Africa. Health systems across the world were challenged by COVID-19^12^ and had to rapidly adapt to mitigate negative impacts. Whilst various strategies were put in place for TB infection control, retention in care and case finding,^13,14^ reports on the adoption of integrated TB and COVID-19 testing to improve case finding are very limited.^15^ Our literature search revealed one study from India in which an integrated TB/COVID-19 algorithm was adopted.^16,17^ All patients with influenza-like symptoms were tested for COVID-19 and those eligible for a COVID-19 test were screened for TB if the individual had any of the following: (1) risk factors for TB (e.g. close contact, diabetes, or elderly), (2) a negative COVID-19 test and symptoms persisting for > 14 days and (3) a positive COVID-19 test and any of: fever > 14 days, cough > 14 days, weight loss and night sweats. In contrast, the algorithm adopted in South Africa used HIV status in combination with the presence and duration of symptoms to determine eligibility for TB testing. The need for integrated screening has been acknowledged,^18^ and further documentation of algorithms implemented and their outcomes would be helpful.

While the decision to offer TB testing was at the discretion of the clinician, our algorithm prompted the clinician to initiate TB testing in the presence of cough of any duration, fever, night sweats, or weight loss if HIV infected, or cough for greater than two weeks, fever, night sweats, or weight loss if HIV uninfected. Very few patients had symptoms of greater than two weeks, possibly as these patients were not screened positive for COVID-19, but went directly to TB services, and so TB testing would have been predominantly indicated amongst patients with HIV.

Our findings showed that approximately 9% (*n* = 79) of patients who screened positive for COVID-19 were tested for TB. Only 24% of those who were HIV positive and presented with a cough or fever received a TB test. Given that our study collected data from the early phases of the model’s implementation, we suspect that there was so much focus on COVID-19 that TB continued to be neglected despite the algorithm. The model was faced with initial implementation challenges such as healthcare worker confusion around when a patient should be tested for both COVID-19 and TB and number of samples to send. Providing clear standard operating procedures for joint sample collection would help in avoiding this confusion in the future. It would also be useful for future research to assess the outcomes of an integrated model, such as this changes several months into its implementation.

Data extracted from the District Health Information System for these two facilities for the period May 2020 to July 2020 show on average a 11% decline in primary head count for those older than 20 years and 63% decline in TB testing when compared with data for the same period in the previous year (a decline from 1010 to 370 TB investigations done). Using the same data periods, we also find a 59% decline in positive TB cases (a drop from 91 to 37 positive TB tests). Our study shows that TB testing during COVID-19 continued at these two facilities. The reduction in TB testing recorded at the onset of COVID-19 is most likely explained by movement restriction and patients’ fear of contracting COVID-19^19,20^ and decreased prioritisation of TB case finding. Our results show that the model was feasible and helped ensure that TB testing in facilities was maintained, albeit at lower levels than historically expected.

Our study has a number of limitations. Our results cannot be generalised, as they represent only two purposively selected facilities. Implementation of fidelity and outcomes may vary across facilities in which the model was implemented. There are a large amount of missing data, as clinical record forms were located for only 846 of 2217 patients entering the orange zone; however, there was no clear pattern of missingness across facilities, suggesting that ascertainment bias was limited. Some records were also poorly captured on the clinical forms. For example, symptom duration was only captured in 40% of the forms. Missing data are a common challenge when using clinical records; however, we have described outcomes of the model as implemented in programmatic conditions which is, therefore, likely scalable. Thirty-five percent of patients had unknown HIV status on presentation, and although the algorithm directed them to HIV-testing services, the results were not available to us.

In conclusion, this study demonstrates the outcomes of a simple and feasible model for integrating TB testing services into COVID-19 care pathways at primary care facilities. Integration of TB and COVID-19 screening and testing should be scaled up, and the results of such scale up were documented.

This material is based upon work supported by the United States President’s Emergency Plan for AIDS Relief (PEPFAR) through the United States Agency for International Development (USAID) under Cooperative Agreement number 72067418CA00023 to the Anova Health Institute. Anova Health Institute is a USAID funded PEPFAR district support partner in South Africa. As such, Anova provides technical assistance at public sector facility level and directs service delivery via healthcare workers employed by Anova. The contents are the responsibility of Anova Health Institute and do not necessarily reflect the views of USAID or the United States Government.
